# Aging and Resilience During Urbanization: A Case Study in a Villagers’ Resettlement Community

**DOI:** 10.1093/geroni/igaf122.4300

**Published:** 2025-12-31

**Authors:** Lingyi Shen

**Affiliations:** Betty Irene Moore School of Nursing, University of California, Davis, Sacramento, California, United States

## Abstract

Kunshan is a county-level city near Shanghai, China. Once a poor region reliant on agriculture, this area has undergone rapid industrialization and urbanization since the 1980s. This resulted in many farmlands being replaced and the emergence of various villagers’ resettlement communities. Local villagers who are Chinese Baby Boomers faced the dual challenge of population aging and urban transformation. This study explores the aging experience of older adults in one of those villagers’ resettlement communities, twenty years after the relocation, investigating their resilience and vulnerabilities emerged in the process. Over a four-month period, ethnographic research was conducted with participant observations, in-depth interviews, as well as oral history interviews focusing on older villagers’ life trajectories and their evolving relationships with different spaces and social organizations during urbanization. Data was analyzed using the grounded theory approach. Three factors are identified that contribute to older villagers’ resilience: 1) adaptive capacities derived from cumulative adversity in their life course; 2) financial and administrative support from village as a collective community of interests; 3) social and religious networks retained from previous rural livelihoods. However, limited education constrains their capacity to articulate their needs despite the presence of third-party services offered within the community. Disparities on social welfare still exist. Despite older villagers’ relatively high satisfaction towards their experience, long-term sustainability of this way of aging is of concern when younger generations are gradually losing the resilience acquired by their parents and grandparents.

